# Intraocular pressure changes and corneal biomechanics after hyperopic small-incision lenticule extraction

**DOI:** 10.1186/s12886-020-01384-2

**Published:** 2020-04-05

**Authors:** Dan Fu, Meiyan Li, Michael C. Knorz, Shengsheng Wei, Jianmin Shang, Xingtao Zhou

**Affiliations:** 1 Department of Ophthalmology, Eye and ENT Hospital of Fudan University, No. 83 FenYang Road, Shanghai, 200031 China; 2 NHC Key Laboratory of Myopia (Fudan University), Shanghai, China; 3 Shanghai Research Center of Ophthalmology and Optometry, Shanghai, China; 4 Medical Faculty Mannheim of the University of Heidelberg, Mannheim, Germany; 5 Xi’An Forth Hospital, Shanxi, China

**Keywords:** Hyperopia, Small-incision lenticule extraction, Intraocular pressure, Corneal biomechanics

## Abstract

**Background:**

We aimed to compare the intraocular pressure (IOP) measurements by a dynamic Scheimpflug analyzer (Corvis ST), a non-contact tonometer, and an ocular response analyzer after hyperopic small-incision lenticule extraction (SMILE).

**Methods:**

Thirteen patients who underwent hyperopic SMILE in one eye were enrolled prospectively. IOP and corneal biomechanical parameters were measured preoperatively and at 1 week, 1 month, and 3 months postoperatively with a non-contact tonometer (IOP_NCT_), Corvis ST (biomechanical corrected IOP [bIOP]), and ocular response analyzer (Goldmann-correlated intraocular pressure [IOPg] and cornea compensated IOP [IOPcc]). A linear mixed model was used to compare the IOPs and biomechanical values among methods at each time point.

**Results:**

IOP_NCT_, IOPg, and IOPcc dropped significantly after surgery, with the amplitude being 3.15 ± 0.48 mmHg, 5.49 ± 0.94 mmHg, and 4.34 ± 0.97 mmHg, respectively, at the last follow-up visit. IOP_NCT_ decreased by 0.11 ± 0.06 mmHg per μm of excised central corneal thickness. bIOP did not change significantly after surgery. Preoperatively, no difference was found among the four measurements (*P* > 0.05). Postoperatively, IOP_NCT_ and bIOP were higher than IOPg and IOPcc. bIOP was independent of cornea thickness at last follow-up visit, whereas it correlated significantly with corneal biomechanics similar to the other three IOP values.

**Conclusion:**

bIOP is a relative accurate measure of IOP after hyperopic SMILE.

## Background

Cornea refractive surgery corrects the refractive error by removing a part of the corneal tissue, and consequently changes both the corneal shape and corneal biomechanics. Previous studies have identified this procedure to change the intraocular pressure (IOP) measurements [1, 2]. Among the nearly 10 million refractive procedures performed, it is estimated that more than 200,000 eyes may be at risk of a missed glaucoma diagnosis based on a conservative 2% incidence of glaucoma [3]. It is generally accepted that IOP measurements falsely decrease after corneal myopic refractive surgery. This phenomenon is observed after photorefractive keratectomy (PRK), laser in situ keratomileusis (LASIK), and myopic small incision lenticule extraction (SMILE) [1, 4].

The principle of hyperopic correction, which differs from that of myopic correction, is to make the central cornea steeper. This is achieved with SMILE by creating a concave lenticule that is thinnest in the central area. Liu [5] reported that hyperopic SMILE can cause more distortion of collagen fibril formation than myopic SMILE in animal models, and therefore, changes in IOP measurements after hyperopic SMILE may be different from that measured after myopic SMILE. Schallhorn et al. [4] reported that hyperopic ablations (both PRK and LASIK) cause lower IOP measurements, smaller in magnitude than that calculated after myopic ablations, and that this decrease in IOP was weakly correlated with preoperative spherical equation after hyperopic LASIK but not hyperopic PRK. Because of the absence of flap in SMILE and the use of a different laser, it would be of great interest to explore the IOP changes induced by hyperopic SMILE.

The current study aimed to explore the effect of hyperopic SMILE on IOP assessment using different measurement methods.

## Methods

### Subjects

Thirteen patients with hyperopic (13 eyes) were enrolled prospectively between March 2017 and June 2018 at the Eye and ENT Hospital of Fudan University (Table 1). Approval was obtained from the institutional ethics committee, and all patients signed informed consent. All procedures adhered to the tenets of the Declaration of Helsinki.

**Table 1:** Baseline information of enrolled patients

	Hyperopia group	(range)
Age (y)	32.8 ± 9.0	(18–45)
Male (%)	3/13	/
Spherical diopter (D)	4.17 ± 1.55	(2.00–6.00)
Cylinder (D)	−0.90 ± 0.75	(−2.25–0.00)
CCT (μm)	546.7 ± 25.3	(507.0–601.0)
Km (D)	42.26 ± 1.12	(40.60–44.70)
Lenticule thickness (μm)	89.0 ± 24.0	(46.0–132.0)

*CCT* central corneal thickness, *Km* mean keratometry

Inclusion criteria were as follows: age ≥ 18 years; sphere + 2 to + 6.0 diopters (D), with astigmatism up to 3.0 D; the difference between manifest and cycloplegic refraction was no more than 1.0 D.

Patients with abnormal topography, dilated pupil size less than 7 mm, history of intraocular surgery, and glaucoma were excluded.

Preoperative examinations included slit lamp examination, objective and subjective refraction assessments, uncorrected distance visual acuity (UDVA) measurement, corrected distance visual acuity (CDVA) measurement, corneal tomography with a rotating Scheimpflug camera (Pentacam, Oculus, Wetzlar, Germany), and fundus examination.

### IOP and corneal biomechanics measurement

**IOP**_**NCT**_**(non-contact IOP):** The non-contact tonometer (TX-20, Canon, Tokyo, Japan) was used. One average value was automatically calculated from 3 measurements. Reproducibility of measurements was identified previously [6].

#### bIOP (biomechanical corrected IOP)

The Corvis ST (Corneal Visualization Scheimpflug Technology instrument; Oculus, Wetzlar, Germany) is a Scheimpflug-based dynamic corneal tonometer, which incorporates the corneal biomechanics and IOP parameters. The system uses an algorithm to calculate biomechanically corrected IOP (bIOP) and compensates for changes in corneal thickness and stiffness [7].

#### IOPg (Goldmann-correlated IOP), IOCcc (cornea compensated IOP), CRF (corneal resistance factor), and CH (corneal hysteresis)

These four values were derived from ocular response analyzer (ORA; Reichert Ophthalmic Instruments, Depew, NY, USA). ORA uses an air puff to deform the cornea. Because of its viscoelastic nature, the cornea resists the air puff, resulting in different values for the inward and outward flexing, which is termed as corneal hysteresis (CH). The corneal resistance factor (CRF) represents the resistance of cornea [8]. Each eye was measured 4 times, and only measurements with a waveform score greater than 5 were used for further analysis.

Two corneal biomechanical parameters derived from Corvis ST were analyzed. A1 Time (first applanation time), and HC DA (deformation amplitude, the largest anterior- posterior displacement of the cornea apex at the highest concavity phase) were the most repeatable and reproducible parameters [9]. SP-A1 (resultant pressure [adjusted pressure at A1 (adj AP1) – biomechanically compensated IOP (Biop)] divided by deflection amplitude at A1) was a new parameter which was used to describe the cornea stiffness, higher values of which meant a more stiff cornea [2].

All measurements were performed by the same examiner (FD) to decrease inter-observer variability and were taken at approximately the same time of day.

### Surgical techniques

All surgeries were performed by the same surgeon (ZXT). After standard sterile draping, all patients were treated with the VisuMax laser (Carl Zeiss Meditec AG, Jena, Germany, version 3.1) with repetition rate of 500 kHz and pulse energy of 30 nJ. The following settings were used for hyperopic SMILE: the cap diameter was 8.8 mm and the thickness 120 μm; the optical zones ranged between 5.3–6.3 mm, with a 2-mm transition zone; a single 2.0-mm side cut was made at the 12 o’clock position with an angle of 90°.

The detailed steps of SMILE have been described previously [10]. Total suction time was approximately 35 s. After lenticule scanning, the surgeon used a splitter to separate the upper interface, following the lower lenticule interface separation. The lenticule was then removed by superior incision. Thereafter, the surgeon examined the cornea with a built-in slit lamp to detect whether parts of the lenticule remained. One drop of prednisolone and levofloxacin was instilled at the end of the surgery.

All surgeries were performed successfully, with no intraoperative or postoperative complications.

Postoperatively, the patients were instructed to use fluorometholone eye drops 8 times a day, and to reduce the usage frequency by 1 every 3 days (totally 24 days). Artificial tears were prescribed for 3 to 4 weeks, for use as needed.

### Follow-up

Patients were examined at 1 week, 1 month, and 3 months postoperatively. At each follow-up visit, the visual acuity, subjective refraction, corneal topography, and IOP measurements were performed using three devices.

### Statistical analyses

All data were recorded and analyzed using SPSS (version 22, IBM Corp, USA). First, the *Kolmogorov–Smirnov* test was used to check the normality of data. Linear mixed-model analysis of variance with post hoc least significant difference multiple comparisons were used to compare the postoperative IOP measurements between different visits and different methods at the same visit. The *Spearman rank* correlation was used to assess the corneal biomechanical parameters obtained from the Corvis ST and to determine potential postoperative factors affecting the postoperative IOP measurements. *P* <  0.05 was considered statistically significant.

## Results

**Fig. 1:**
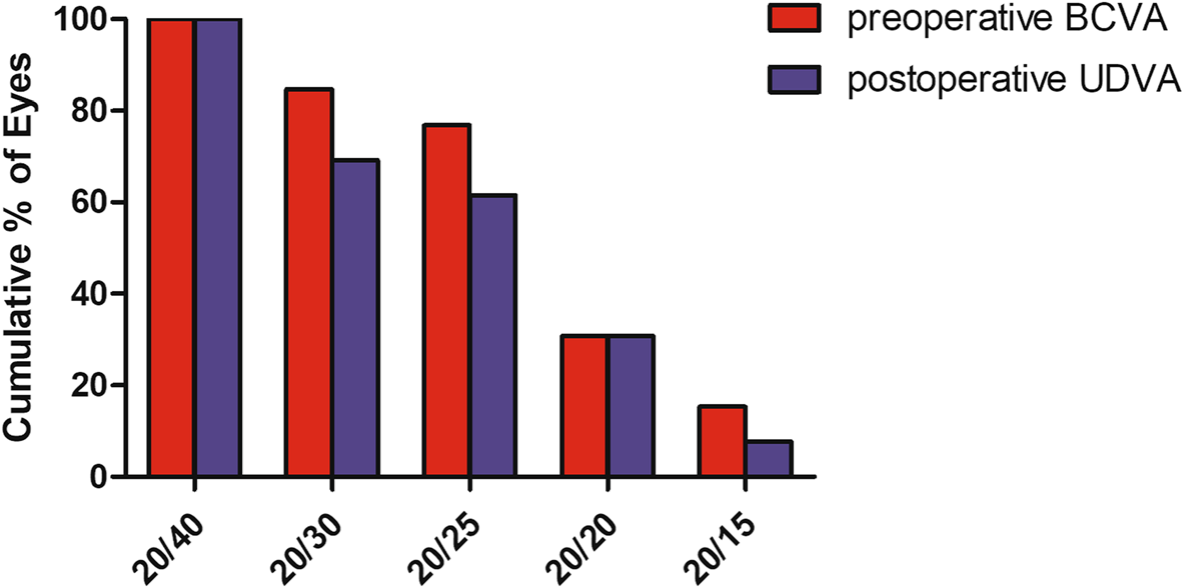
Preoperative corrected visual acuity and postoperative visual acuity at the last visit

All patients completed the 3-months follow-up visit. The safety index (postoperative CDVA/preoperative CDVA) was 0.96 ± 0.12, and the efficacy index (postoperative UDVA/preoperative CDVA) was 0.93 ± 0.14 at the last visit. (Fig. 1) The refraction at each visit time is shown in Table 2**.**

**Table 2:** Mean refraction(D) at each visit time

	Pre-op	Post-1w	Post-1 m	Post-3 m
Spherical diopter	4.2 ± 1.5	− 0.1 ± 1.4	0.2 ± 1.5	0.5 ± 1.0
cylinder	−0.9 ± 0.7	−0.5 ± 0.5	−0.5 ± 0.5	−0.5 ± 0.4

*D* diopter

### Changes in IOP measurement

The IOP values at different points of time are shown in Table 3. Preoperatively, no difference was found among the measurements. At 1 week postoperatively, the IOP_NCT_ was 2.52 ± 1.11 mmHg higher than IOPg (*P* = 0.04); bIOP was 2.32 ± 0.85 mmHg higher than IOPg (*P* = 0.02); IOP_NCT_ and bIOP values showed no difference. At 1 month postoperatively, bIOP was 3.60 ± 0.89 mmHg higher than IOPg (*P* = 0.004) and 3.32 ± 0.86 higher than IOPcc (*P* = 0.005), and IOP_NCT_ was 2.56 ± 0.50 mmHg higher than IOPg (*P* = 0.001). No difference was found between IOP_NCT_ and bIOP values. At 3 months postoperatively, bIOP was the highest IOP value (IOP_CC_: Δ = 3.29 ± 0.63 mmHg, *P* = 0.001; IOPg: Δ = 3.68 ± 0.91 mmHg, *P* = 0.003; IOP_NCT_: Δ = 2.13 ± 0.70 mmHg, *P* = 0.01). IOPg and IOPcc values showed no difference at all postoperative visits.

**Table 3:** Mean IOP values (mmHg) of the four methods at each time point

	pre	Post-1w	Post-1 m	Post-3 ms	P
IOP_NCT_	15.40 ± 3.20	14.73 ± 3.74	13.87 ± 3.96	12.08 ± 2.83	0.006
bIOP	15.77 ± 4.18	14.21 ± 1.87	14.30 ± 1.76	14.13 ± 1.61	0.878
IOPg	15.23 ± 4.84	11.75 ± 3.56	10.36 ± 3.19	10.51 ± 3.83	< 0.001
IOP_CC_	14.62 ± 3.94	11.54 ± 1.07	10.46 ± 2.83	10.90 ± 2.80	< 0.001
P	0.928	0.045	< 0.001	< 0.001	

*IOP*_*NCT*_ non-contact intraocular pressure, *biop* biomechanical corrected intraocular pressure, *IOPg* Goldmann-correlated intraocular pressure, *IOPcc* cornea compensated intraocular pressure

Except bIOP, compared with the preoperative values, the other three measurements were lower postoperatively. IOP_NCT_ remained stable from before surgery to 1 month after the surgery (postoperative 1 month vs postoperative 3 months, Δ = 1.85 ± 0.82 mmHg, *P* = 0.04), and decreased 3.15 ± 0.48 mmHg at postoperative 3 months compared with the preoperative values (*P* <  0.001; 0.11 ± 0.06 mmHg reduction per micro removed cornea tissue [ΔIOP_NCT_/lenticule thickness]).

**Fig. 2:**
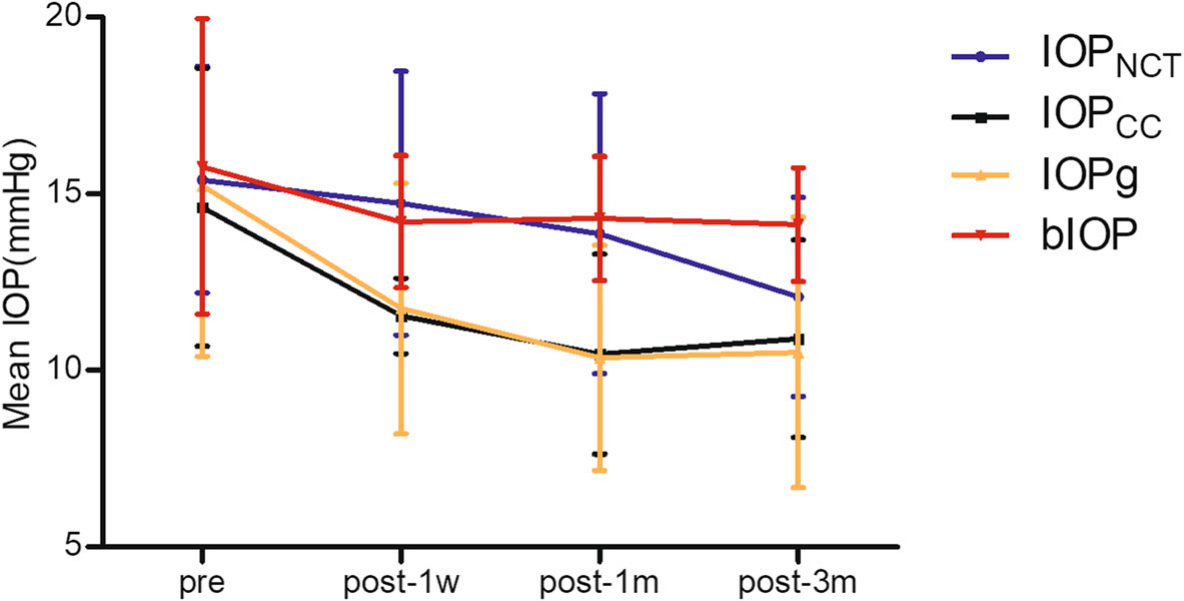
Changes in mean intraocular pressure measurement during postoperative follow-up

Compared with the preoperative values, IOPcc started to decrease at postoperative 1 week (Δ = 2.71 ± 1.04 mmHg, *P* = 0.03), and decreased until 1 month postoperatively (Δ = 4.94 ± 1.25 mmHg, *P* = 0.006). IOPg decreased by 4.30 ± 1.13 mmHg (*P* = 0.007) at 1 week postoperatively and remained stable thereafter. Among all 4 measurements at postoperative 3 months postoperatively, IOPg showed the greatest difference between pre- and postoperative values (Δ = 5.49 ± 0.94 mmHg, *P* = 0.001) (Fig. 2).

### Changes in corneal biomechanical and correlation analysis

The biomechanical parameters from ORA and Corvis ST are shown in Table 4**.** CRF and SP-A1 dropped significantly after surgery (*P* <  0.05). CH, A1 Time and HC DA showed no significant difference among the follow-up visits.

**Table 4:** Corneal biomechanical parameters measured using the ocular response analyzer and Corvis ST

	Pre-op	Post-1w	Post-1 m	Post-3 ms	P
CH	11.47 ± 1.47	10.88 ± 1.19	11.05 ± 1.95	11.12 ± 1.14	0.13
CRF	11.25 ± 2.22	9.71 ± 1.64	10.15 ± 2.33	9.51 ± 1.87	.001
A1 Time (ms)	7.10 ± 0.52	6.87 ± 0.24	6.93 ± 0.57	6.88 ± 0.31	0.23
HC DA (mm)	1.03 ± 0.14	1.13 ± 0.14	1.14 ± 0.15	1.11 ± 0.08	0.12
SP -A1	114.87 ± 16.67	93.64 ± 18.00	92.08 ± 20.82	94.03 ± 19.07	0.001

*CH* cornea hysterisis, *CRF* cornea resistance factor, *A1 Time* first applanation time, *HC DA* deformation amplitude, the largest anterior-posterior displacement of the corneal apex at the highest concavity phase, *SP-A1* Resultant pressure [adjusted pressure at A1 (adj AP1) – biomechanically compensated IOP (Biop)] divided by deflection amplitude at A1

Using Spearman analysis, at postoperative 3 months follow-up, HC DA was negatively related with all IOPs (r ranges from − 0.82 to − 0.74, *P* <  0.05). IOPg and IOPcc were correlated with CRF (*r* = 0.68–0.91; *P* <  0.05) and postoperative CCT (*r* = 0.83–0.95; *P* <  0.01). bIOP was independent of preoperative CCT as well as postoperative CCT and correlated with A1 Time (*r* = 0.87; *P* = 0.001) and HC DA (*r* = − 0.74; *P* = 0.01) at the last postoperative follow-up visit. IOP_NCT_ at the last postoperative follow-up visit correlated with homologous CRF, A1 Time, and HC DA as well as preoperative IOP_NCT_ (*r* = 0.86; *P* = 0.001) (Table 5).

**Table 5:** Correlations between IOPs and corneal biomechanical parameters at 3 months visit (r [P])

	IOP_NCT_	bIOP	IOPg	IOPcc
CH	0.48 (0.16)	0.28 (0.44)	0.56 (0.09)	0.22 (0.54)
CRF	**0.75 (0.01)**	0.59 (0.07)	**0.91 (0.001)**	**0.68 (0.03)**
A1 Time (ms)	**0.66 (0.03)**	**0.87 (0.001)**	**0.71 (0.03)**	0.64 (0.06)
HC DA (mm)	**− 0.76 (0.006)**	**− 0.74 (0.01)**	**− 0.82 (0.007)**	**− 0.76 (0.02)**
SP A1	0.49 (0.15)	0.32 (0.37)	**0.72 (0.04)**	0.49 (0.22)
CCT (μm)	0.49 (0.11)	0.32 (0.31)	**0.95 (0.001)**	**0.83 (0.003)**
Km (D)	−0.05 (0.89)	0.29 (0.36)	−0.24 (0.51)	− 0.36 (0.30)

*r* correlation coefficient, *CCT* central cornea thickness at 3 month after surgery, *CH* cornea hysterisis, *CRF* cornea resistance factor, *A1 Time* first applanation time, *HC DA* deformation amplitude, the largest anterior-posterior displacement of the corneal apex at the highest concavity phase, *SP-A1* Resultant pressure [adjusted pressure at A1 (adj AP1) – biomechanically compensated IOP (Biop)] divided by deflection amplitude at A1, *IOPNCT* non-contact intraocular pressure, *biop* biomechanical corrected intraocular pressure, *IOPg* Goldmann-correlated intraocular pressure, *IOPcc* cornea compensated intraocular pressure

## Discussion

Accurate IOP measurement is extremely important for ophthalmologists because false low readings of IOP may delay the diagnosis of ocular hypertension or glaucoma [11]. In this study, we evaluated the effect of hyperopic SMILE on different IOP measurement techniques. To our best knowledge, it is the first report of this kind.

In this study, the average decrease of IOP measurements from pre- to postoperatively ranged from 0.42–5.48 mmHg among the different measurement techniques used. Lee [12] reported that 6 months postoperatively, IOP_NCT_ decreased by 2.04 ± 1.44 mmHg after myopic transepithelial PRK and by 2.63 ± 1.60 mmHg after myopic femtosecond-LASIK. Li [13] demonstrated that ΔIOP_NCT_ per micrometer of ablated tissue after 6 months postoperatively was 0.05 ± 0.02 mmHg in myopic SMILE group, and 0.05 ± 0.03 mmHg in myopic femtosecond-LASIK group, which was lesser than 0.11 ± 0.06 mmHg calculated in the present study. Reinstein et al. [14] found that postoperative tensile strength was greatest after SMILE, followed by PRK, and was lowest after LASIK. Thus, different corneal stiffness impairments may partially account for the different IOP reduction among surgeries. In addition, epithelium preservation and flap-free procedures may result in difference in pressure resistance. Moreover, the hyperopic lenticule, different from myopic ones, is thinnest at the center and causes less thinning of the central cornea. It may lead to different wound healing processes, although the direct relationship between would healing and IOP measurement remains to be identified [15].

The present study used four IOP measurement methods; three of them attempted to correct for the biomechanical changes of the cornea caused by corneal refractive surgery. We found that bIOP (biomechanical corrected IOP, measured with the Corvis ST) most closely matched the approximate preoperative IOP values, whereas the other three estimated IOP values were lower after hyperopic SMILE. Similar results were observed after myopic LASIK and myopic SMILE [16, 17]. Lee [12] also reported that bIOP values remained unchanged after myopic LASIK and PRK. Previous studies have demonstrated that CCT can influence IOP measurement. Liu et al. [18] reported that IOP readings may have a 2.87-mmHg range because of CCT variations, but have a much larger 17.26-mmHg range because of changes in corneal biomechanical properties alone. Biomechanical properties therefore seem to have a much greater effect on IOP than CCT. In this study, we found that both cornea biomechanics and CCT correlated with the IOP values. However, we found no correlation between pre and postoperative CCT values for bIOP; they were similar after both hyperopic and myopic SMILE, indicating it is a reliable assessment method postoperatively.

In this study, IOPg and IOPcc remained constant at all postoperative follow-up visits, and both values correlated with CCT as well as cornea biomechanics. This was consistent with the results of Mollan, [19], but contrary to that reported by Sullivan-Mee [20], who reported that IOPcc was higher than IOPg. Different types of ocular pathologies may be responsible for this discrepancy. Sullivan-Mee [20] measured IOPg and IOPcc in patients with suspected or diagnosed glaucoma, whereas Mollan examined patients with keratoconus and a control group. IOPg, an average value of inward and outward pressures, is considered identical with that observed with Goldmann applanation tonometry [21]. IOPcc is affected to a less degree by variations in corneal thickness and corneal biomechanical properties. The present results indicate that IOPg and IOPcc show no difference for hyperopic SMILE related IOP changes.

IOP_NCT_ was the most commonly used clinical parameter. In this study, IOP_NCT_ decreased by 3.15 ± 0.48 mmHg after hyperopic SMILE, greater than bIOP, but less than IOPg and IOPcc. Although Wolfs et al. reported a positive correlation of IOP with CCT, [22] we found no significant correlation between preoperative CCT and IOP_NCT_ values in this study. In addition, IOP_NCT_ at last follow-up visit correlated with preoperative IOP_NCT_ and some corneal biomechanical properties, suggesting that corneal biomechanics but not CCT may be of greater importance when predicting IOP_NCT_ after hyperopic SMILE. Additional studies are warranted to further validate these findings.

This study has several limitations. The study lacks corresponding measurements with Goldmann applanation tonometer, which is considered the gold standard reference for IOP measurements. The enrolled cases had astigmatism, which did not lead to pure hyperopia correction, though cornea thickness and biomechanics are major factors affecting IOP and mixed astigmatism might probably have some effect on the results. In addition, the sample size in the current study is small, and a larger sample size and longer study duration are needed.

## Conclusion

In conclusion, IOP_NCT_ decreased after hyperopic SMILE, and its value correlates with preoperative IOP_NCT_ as well as corneal biomechanical properties. bIOP seems to be an accurate parameter to assess postoperative IOP.

## Data Availability

The datasets used and analyzed during the current study are available from the corresponding author on reasonable request.

## Abbreviations

IOP: Intraocular pressure
SMILE: Small-incision lenticule extraction
IOP_NCT_: Non-contact tonometer
bIOP: Biomechanical corrected IOP
ORA: Ocular response analyzer
IOPg: Goldmann-correlated intraocular pressure
IOPcc: Cornea compensated IOP
LASIK: Laser in situ keratomileusis
PRK: Photorefractive keratectomy
UDVA: Uncorrected distance visual acuity
CDVA: Corrected distance visual acuity
CH: Corneal hysteresis
CRF: Cornea resistant factor
A1 Time: First applanation time
HC DA: Deformation amplitude, the largest anterior- posterior displacement of the cornea apex at the highest concavity phase
SP- A1: Resultant pressure [adjusted pressure at A1 (adj AP1) – biomechanically compensated IOP (Biop)] divided by deflection amplitude at A1
